# Normative Values and Psychometric Properties of EQ-5D-Y-3L in Chilean Youth Population among Different Weight Statuses

**DOI:** 10.3390/ijerph20054096

**Published:** 2023-02-24

**Authors:** Miguel Angel Perez-Sousa, Pedro R. Olivares, Rocio Carrasco-Zahinos, Antonio Garcia-Hermoso

**Affiliations:** 1 Department of Physical Education, Faculty of Education, University of Córdoba, 14071 Cordoba, Spain; 2 Epidemiology of Physical Activity and Fitness Across Lifespan Research Group, University of Seville, 41004 Seville, Spain; 3 Faculty of Education, Psychology and Sport Sciences, University of Huelva, Avenida de las Fuerzas Armadas s/n 21007, 21004 Huelva, Spain; 4 Instituto de Actividad Fisica y Salud, Universidad Autonoma de Chile, Talca 3460000, Chile; 5 Navarrabiomed, Hospital Universitario de Navarra (HUN), Universidad Pública de Navarra (UPNA), IdiSNA, 31008 Pamplona, Spain; 6 Physical Activity, Sport and Health Sciences Laboratory, University of Santiago de Chile, Santiago 9170020, Chile

**Keywords:** quality of life, normative values, validity, children

## Abstract

Background: This study aimed to provide population norms among children and adolescents in Chile using the EQ-5D-Y-3L questionnaire and to examine its feasibility and validity among body weight statuses. Methods: This was a cross-sectional study in which 2204 children and adolescents (aged 8–18 years) from Chile completed a set of questionnaires providing sociodemographic, anthropometric and health-related quality of life (HRQoL) data using the five EQ-5D-Y-3L dimensions and its visual analogue scale (EQ-VAS). Descriptive statistics of the five dimensions and the EQ-VAS were categorized into body weight status groups for the EQ-5D-Y-3L population norms. The ceiling effect, feasibility and discriminant/convergent validity of the EQ-5D-Y-3L were tested. Results: The dimensions of the EQ-5D-Y-3L questionnaire presented more ceiling effects than the EQ-VAS. The validity showed that the EQ-VAS could discriminate among body weight statuses. However, the EQ-5D-Y-3L index (EQ-Index) demonstrated a non-acceptable discriminant validity. Furthermore, both the EQ-Index and the EQ-VAS presented an acceptable concurrent validity among weight statuses. Conclusions: The normative values of the EQ-5D-Y-3L indicated its potential use as a reference for future studies. However, the validity of the EQ-5D-Y-3L for comparing the HRQoL among weight statuses could be insufficient.

## 1. Introduction

Health-related quality of life (HRQoL) has been defined as a multidimensional concept beyond somatic indicators, including physical, psychological, social and functional aspects of self-assessment of the individual’s health [1]. The increases in chronic illness in children and adolescents [2] have framed HRQoL assessment as of significant interest to public health. This fact was indicated by the US Food and Drug Administration and the pharmaceutical industry, who recognize the need for assessing HRQoL in pediatric and adolescent patients to determine the effects of pharmacological treatments to complete the biomedical perspective [3].

HRQoL is measured via self-report or proxy report from a standardized questionnaire that includes different dimensions. The questionnaire provides a generic health perception allowing comparisons between different populations and conditions and also an econometric result that could be used in cost–utility analysis for economic evaluation [4].

The main HRQoL questionnaires for children and adolescents, including the PedsQL [5], Kidscreen [6], and EQ-5D-Y-3L [7], have culturally adapted their versions for most countries.

The EQ-5D-Y-3L is a widely used questionnaire with five dimensions of health (“mobility,” “looking after myself,” “doing usual activities,” “having pain or discomfort,” and “feeling worried, sad or unhappy”) and three levels of response indicating the severity of health problems in the participant, providing 243 possible health states [8]. The EQ-5D-Y-3L has been translated and adapted to Spanish and presents acceptable validity and reliability [7]. This questionnaire is also used in Latin American countries [9], but to our knowledge, there are scant normative data for this region.

Within the broad spectrum of childhood diseases, obesity takes up a prominent position due to its prevalence and effects on physical and psychological health [10,11]. One of the principal components of chronic illness in children and adolescents living in Latin American countries is overweight and obesity, which has grown continuously in the last decade [12]. In this respect, previous studies have shown an inverse relationship between body mass index (BMI) and HRQoL. For example, Perez-Sousa et al. [13] found that overweight and obese Spanish children showed a lower HRQoL than their normal-weight counterparts. Garcia-Rubio et al. [14] showed that overweight and obese children and adolescents had a reduced HRQoL compared to healthy children in a cross-sectional study carried out in Chile. However, several studies have presented a muddled relationship between excess body weight and HRQoL. For example, Petersen et al. [15] found a similar HRQoL in children with obesity and normal weight, and Liu et al. [16] only found a lower HRQoL for the social dimension in overweight/obese children compared with healthy-weight children after controlling for gender, age, school type, parental education and family income. In general terms, the studies emphasize that the lack of differences found may be due to cultural and/or socioeconomic characteristics. However, we hypothesize that the questionnaire cannot discern different health perceptions between weight status due to a lack of knowledge on performance regarding psychometric properties of the questionnaire in these subgroups.

Population norms are essential to characterize the study population, interpret research results, and compare studies. Furthermore, this action allows comparison of results from the general population or people with specific health characteristics in order to develop primary physician care standards [17]. However, Chile lacks studies on normative values of HRQoL in children and adolescents from general and specific populations using the EQ-5D-Y-3L questionnaire. Thus, based on the current evidence and the importance of screening for HRQoL within children and adolescents, we aimed to provide normative population values for HRQoL and examine the feasibility and convergent/discriminant validity among Chilean children and adolescents with different weight status using the EQ-5D-Y-3L.

## 2. Methods

### 2.1. Study Design and Participants

A cross-sectional analysis was conducted using data collected from 2204 Chilean children and adolescents aged 8–18 years from the general population. We recruited 3150 participants from primary and secondary schools in Chile and 2204 of these finally agreed to participate in the interviews. We requested the participation of eight schools (four primary and four secondary), with each providing access to four or five sections of different grades. According to the design, participants who met the following inclusion criteria formed our target group: children and adolescents aged 8–18 years; knowledge of the Spanish language; present on the day of the test; and gave their informed consent (subjects and parents or legal tutors).

Before data collection, the parents were informed of the methodology and objectives of the study via an official letter written by the researchers that included an informed consent form. The study was approved by University of Santiago Ethics Committee (code 938).

### 2.2. Procedure

The data were collected by two experienced research group members using direct administration in small groups (10–12 children per group). The survey duration varied from 5 min for children aged 8–12 years to 3 min for students aged 13–18 years. Each respondent was assigned a code for confidentiality and to facilitate data analysis. A phone number and email address were provided to respondents to address any concerns that may arise at any time.

### 2.3. Measures

#### 2.3.1. Sociodemographic Information

A core set of questions on essential sociodemographic characteristics (age, gender and year of schooling) and HRQoL and subjective health measures were included. For anthropometric data, weight and height were assessed with the participants standing barefoot in minimal clothing. The instrument used was a Seca 769 (Seca, Hamburg, Germany) scale with a portable Seca 220 stadiometer (accuracy of 0.1 cm; Seca, Hamburg, Germany) placed on a rigid wall. BMI was calculated as the body weight divided by the squared height (kg/m^2^). Individuals were classified into four categories according to their BMI as follows: (0) underweight, (1) normal weight, (3) overweight and (4) obese, as indicated by Cole et al. [18].

#### 2.3.2. Health-Related Quality of Life

The EuroQol group developed a tool with five dimensions (the EQ-5D) to quantify HRQoL. The dimensions are mobility, self-care, usual activities, pain or discomfort and anxiety or depression. The instrument also includes a visual analogue scale (EQ-VAS), which is anchored at 100 (best imaginable health) and 0 (worst imaginable health). Most recently, the EuroQol group implemented a version for children and adolescents between the ages of 8 and 18 years, called the EQ-5D-Y-3L [7]. The five questions are whether children have problems with walking, looking after themselves, doing their usual activities, have pain or discomfort and feel worried, sad or unhappy, to which they could respond with “no problems,” “some problems” and “a lot of problems.” The EQ-5D-Y-3L offers a state of health that can be converted into a unique index (EQ-Index) by applying a formula that attributes different weights to each dimension’s levels. The anchor points or references of the questionnaire are 0 (death) and 1 (perfect health). We used the formula to assess adult health status in Spain [19]. This procedure has already been applied in similar studies [20,21]. The reliability and validity of the Spanish version of the EQ-5D-Y-3L has been confirmed [7] and the EQ-VAS allows subjects to assess their health status from 0 (worst) to 100 (best).

#### 2.3.3. Statistical Analysis

A descriptive analysis using the means ± standard deviation (SD) for continuous variables and frequency distribution for categorical variables was used to obtain the characteristics of the sample.

##### Population Norms

The EQ-5D-Y-3L population norms were derived from the data given by the general population sample. Analysis of the EQ-5D-Y-3L population norms followed the standardized method recommended by the EuroQol group [22].

##### Feasibility

We computed the proportion of children not answering to a few (i.e., partially incomplete questionnaire) or all dimensions (i.e., incomplete questionnaire) of the EQ-5D-Y-3L.

##### Ceiling Effect

The proportions of children reporting “no problems” were calculated for each descriptive system dimension. We also computed the children reporting “no problems” ratio in all five dimensions (11111). We hypothesized that normal-weight children would report a higher ceiling effect than their counterparts.

##### Discriminant and Convergent Validity

The discriminant validity of the EQ-5D-Y-3L was examined by comparing the HRQoL profiles of the different weight status groups (underweight, normal weight, overweight and obesity). The level of problems reported in each EQ-5D-Y-3L dimension per group was compared using Fisher’s exact test rather than the chi-square test because some cells were sparsely populated. Post hoc analysis using the Kruskal–Wallis H test indicated which groups were significantly different from each other. Following studies in overweight and obese children [23,24], we assumed that complaints of health problems would be more common among underweight, overweight and obese children and that these individuals would therefore have lower scores on the EQ-5D-Y-3L dimensions and EQ-VAS than normal-weight children. The convergent validity of the EQ-5D-Y-3L was examined by correlating the EQ-Index with the EQ-VAS through Spearman’s rho correlation. The correlation coefficient (*ρ*_s_) was interpreted as follows: small, 0.10–0.29; moderate, ≥0.30–0.49; strong, ≥0.50 [25].

Convergent validity is the ability of the scores to correlate with other measures that assess a similar construct. In contrast, discriminant validity examines the relationships of scores obtained from similar but different constructs [25].

## 3. Results

Table 1 shows the characteristics of the general population sample. Overall, a total of 2204 children and adolescents responded to the set of questions in the EQ-5D-Y-3L. The sample distribution was higher for females (1313 ± 59.6%) than males (891 ± 40.4%). The proportions among weight status groups were dissimilar, with the majority of respondents in the normal-weight group (43.5%). The mean ± SD of the EQ-Index by gender, age group and weight status group are also presented.

The frequency of reported problems by weight status group is shown in Table 2. Fisher’s exact and Kruskal–Wallis analysis showed nonsignificant differences (*p* > 0.05) in the distribution of problems for each dimension of the EQ-5D-Y-3L; therefore, there were no differences in problems reported for HRQoL among underweight, normal-weight, overweight and obese children over the EQ-5D-Y-3L dimensions. Thus, the discriminant validity of the descriptive system appeared to be lower and was unable to discern problems among children and adolescents with different weight status. In contrast, there were statistically significant differences in HRQoL reported on the EQ-VAS among all weight status groups. The ceiling effect (no problems reported) was relatively higher in the physical dimensions (mobility; looking after myself; doing usual activities), whereas the psychological dimensions (having pain or discomfort; feeling worried, sad or unhappy) showed a lower ceiling effect in all groups.

Finally, convergent validity was examined (Table 3). Spearman’s rho test showed a significant correlation (*p* < 0.001) between all dimensions in all groups and for the EQ-VAS, with the exception of the “looking after myself” and “feeling worried, sad or unhappy” dimensions in the overweight group. The magnitude of the correlation was low in all dimensions and groups, except for “mobility,” “doing usual activities” and “feeling worried, sad or unhappy” in the underweight group.

## 4. Discussion

This study has provided population norms for the EQ-5D-Y-3L questionnaire by using a representative sample of Chilean children and adolescents (*n* = 2204) and has demonstrated the psychometric properties in terms of feasibility and discriminant/convergent validity to determine the instrument’s ability to discern health states among weight status groups.

A strength of this study’s EQ-5D-Y-3L population norms was the neutral context sample with the responses pooled across different weight statuses. To date, this is the first study to present normative data in Chilean children and adolescents using the EQ-5D-Y-3L questionnaire. Other studies in Europe [7] or North America [26] have been conducted in the general population.

The main findings of this study were that the Spanish version of the EQ-5D-Y-3L is a feasible instrument to assess HRQoL in the Chilean population because there were no missing values. The results are consistent with previous research, including a multinational study performed to analyze the validity and reliability of the EQ-5D-Y-3L [7]. Our study identified a higher ceiling effect on the physical dimensions (mobility; looking after myself; doing usual activities) and a lower effect on the psychological dimensions (having pain or discomfort; feeling worried, sad or unhappy) in all groups. This ceiling effect is similar to previous studies in the general population [7]. Furthermore, a previous study that used the EQ-5D-Y-3L with overweight and obese children reported few problems in the majority of dimensions, except for anxiety/depression [27].

Another finding in this study was the scarce discriminant validity of the descriptive system of the EQ-5D-Y-3L between health states across weight status. There were no significant differences in the distribution of problems in each dimension among underweight, normal weight, overweight and obese children. These results are similar to previous studies [7,27,28]. In contrast, we found several reviews that analyzed how overweight and obesity affect children’s HRQoL [29,30]. However, the questionnaires that assessed HRQoL were Kidscreen, PedsQL and KINDL-R. These questionnaires are based on 5–7 levels of response, whereas the EQ-5D-Y-3L only has three levels of response. Moreover, we found other studies where the score from PedsQL or Kidscreen discriminates significant health status among weight status groups. The score for these questionnaires is based on a scale of 0–100, whereas the EQ-5D-Y-3L dimensions are based on a score of 1–3. Nevertheless, our study found that the EQ-VAS was discerned among health states across weight groups. This finding suggests that a scale such as the EQ-VAS, based on 0–100%, may be more accurate in identifying health states than a descriptive system based on three levels of response. This low discriminant validity of the descriptive dimension may be due, first, to the high ceiling effect of this instrument. Second, there is the effect of non-dimensionality of the EQ-VAS, with the descriptive dimension and the EQ-VAS starting from a different scale: the descriptive system is based on five dimensions of the state of health and the EQ-VAS as a percentage of the state of health compared to the best imaginable. Therefore, the EQ-VAS can cover as many different dimensions of health as the respondent interprets, all reduced to a single value. Third, several studies indicate that the response in the EQ-VAS is influenced not only by health status but also by personal characteristics such as age, gender, education and race [31,32,33]. Expanding the severity levels in the EQ-5D-Y-3L can reduce the instrument’s ceiling effects and enhance sensitivity, especially in milder health conditions [34]. Thus, it is probable that discriminant validity will be better using the new EQ-5D-Y-3L-5L instrument [35,36].

The convergent validity of the EQ-5D-Y-3L dimensions for each weight status group showed a significant association with the EQ-VAS, but the magnitude of correlation in general was low. Thus, these results should be considered with caution.

Our study has certain limitations. We did not collect information concerning comorbidities or include other populations, such as hospitalized children or those with chronic diseases. These factors need to be considered when applying normative data in other groups or individuals. Furthermore, this study was observational; thus, we might have missed some confounders. Additionally, the method was self-administration, whereas other studies apply proxy administration. Another limitation is the low prevalence of severity of health problems captured by the instruments used. Although children with overweight or obesity have a lower HRQoL than children with a healthy weight [24], the baseline state of their HRQoL using currently available instruments and assessments starts from a high level, which limits the capture of possible improvements. This fact is determined by the ceiling effect of the questionnaire, which can be observed in the proportion of individuals with severe or large HRQoL problems. Likewise, this ceiling effect has been reported in the EQ-5D-EL-Y with studies on individuals without severe health problems [1]. In fact, the EuroQol group is developing a version of the questionnaire with five response levels (EQ-5D-5L-Y) to obtain greater scaling in certain populations. Therefore, our results should be considered with caution.

The study strengths include the large sample: 2204 Chilean children and adolescents. Moreover, the results of this study provide better understanding and use of the EQ-5D-Y-3L questionnaire in children with obesity and help in deciding whether to use this questionnaire over another and how to interpret the results.

## 5. Conclusions

The Chilean population norms for the EQ-5D-Y-3L reported here can be used as reference values when comparing different weight status groups. Furthermore, the study confirmed its feasibility even though the convergent and discriminant validity of the EQ-5D-Y-3L was insufficient. Consequently, we recommend that the results of future studies using the EQ-5D-Y-3L on children with heterogeneous weight status should be interpreted with caution.

## Figures and Tables

**Table 1:** Participant characteristics.

	Sample *n*, (%)	EQ-Index	
		Mean	SD
**Overall**	*n* = 2204	0.88	0.19
**Sex**			
Male	1313 (59.6)	0.87	0.20
Female	891 (40.4)	0.88	0.17
**Age-groups**			
8–10	894 (44.6)	0.85	0.21
11–13	582 (26.4)	0.87	0.20
14–16	425 (19.3)	0.91	0.14
17–18	213 (9.7)	0.93	0.11
**Weight status groups**			
Underweight	159 (7.2)	0.89	0.17
Normal weight	959 (43.5)	0.88	0.18
Overweight	650 (29.5)	0.88	0.18
Obese	436 (19.8)	0.86	0.22

**Table 2:** Percentage frequency distribution of EQ-5D-Y-3L dimensions and VAS by weight status.

	Underweight (*n* = 159)		Normal Weight (*n* = 959)		Overweight (*n* = 650)		Obese (*n* = 159)			
Dimensions	*n*	*%*	*n*	*%*	*n*	*%*	*n*	*%*	p *	p **
Mobility (walking about)										
No problems	140	88.1	870	90.7	579	89.1	389	89.2	0.727	0.594
Some problems	19	11.9	83	8.7	66	10.2	43	9.9		
A lot of problems	0	0.0	6	0.6	5	0.8	4	0.9		
Looking after myself										
No problems	149	93.7	905	94.4	612	94.2	403	92.4	0.729	0.560
Some problems	10	6.3	47	4.9	35	5.4	30	6.9		
A lot of problems	0	0	7	0.7	3	0.5	3	0.7		
Doing usual activities										
No problems	140	88.1	855	89.2	578	88.9	379	86.9	0.333	0.621
Some problems	19	11.9	98	10.2	68	10.5	49	11.2		
A lot of problems	0	0	6	0.6	4	0.6	8	1.8		
Having pain or discomfort										
No pain or discomfort	107	67.3	675	70.4	446	68.6	303	69.5	0.349	0.879
Some pain or discomfort	50	31.4	258	26.9	192	29.5	117	26.8		
A lot of pain or discomfort	2	1.3	26	2.7	12	1.8	16	3.7		
Feeling worried, sad or unhappy										
Not worried, sad or unhappy	116	73.0	661	68.9	452	69.5	295	67.7	0.223	0.647
A bit worried, sad or unhappy	35	22.0	265	27.6	165	25.4	114	26.1		
Very worried, sad or unhappy	8	5.0	33	3.4	33	5.1	27	6.2		
VAS (mean, SD)	82.9	19.3	78.6	20.7	79.8	20.1	80.6	22.1	0.000 †	0.000 ‡

p *, Fisher’s exact test; *p* **, Kruskal–Wallis H test; †, One-way ANOVA; ‡, Bonferroni post hoc.

**Table 3:** Convergent validity: Spearman’s correlation coefficients.

EQ-5D-Y-3L Dimensions	Underweight	Normal Weight	Overweight	Obese
Mobility (walking about)	−0.336 *	−0.129 *	−0.085 *	−0.095 *
Looking after myself	−0.110	−0.045	−0.013	−0.015
Doing usual activities	−0.330 *	−0.102 *	−0.171 *	−0.114 *
Having pain or discomfort	−0.317 *	−0.160 *	−0.195 *	−0.123 *
Feeling worried, sad or unhappy	−0.206 *	−0.178 *	−0.061	−0.250 *

* *p* < 0.05 for all correlation coefficients.

## Data Availability

Data are available upon request to study authors.
